# Efficient Endolymphatic Sac-Directed Gene Delivery Using AAV8BP2 and Posterior Semicircular Canal Injection

**DOI:** 10.3390/ijms27177884

**Published:** 2026-09-03

**Authors:** Minjin Kang, Michelle J. Suh, Heon Yung Gee, Wade W. Chien, Jinsei Jung

**Affiliations:** 1 Department of Otorhinolaryngology, Severance Biomedical Science Institute, Yonsei University College of Medicine, Seoul 03722, Republic of Korea; mjkang2460@yuhs.ac; 2 Won-Sang Lee Institute for Hearing Loss, Yonsei University, Seoul 03722, Republic of Korea; hygee@yuhs.ac; 3 Bio-Centennial Convergence Institute, Yonsei University, Seoul 03722, Republic of Korea; 4 Department of Otorhinolaryngology, Jeju National University Hospital, Jeju 63241, Republic of Korea; myzetapotential@gmail.com; 5 Department of Pharmacology, Graduate School of Medical Science, Brain Korea 21 Project, Yonsei University College of Medicine, Seoul 03722, Republic of Korea; 6 Section on Inner Ear Therapeutics, Neurotology Branch, National Institute on Deafness and Other Communication Disorders, National Institutes of Health, Bethesda, MD 20892, USA; wade.chien@nih.gov

**Keywords:** AAV8BP2, endolymphatic sac, Pendred syndrome, DFNB4, SLC26A4

## Abstract

Mutations in *SLC26A4*, which encodes the anion transporter pendrin, represent one of the most common genetic causes of hereditary hearing loss, including Pendred syndrome and DFNB4. Pendrin plays a critical role in maintaining ion homeostasis within the inner ear, particularly in the endolymphatic sac (ES), making it an important therapeutic target for gene replacement strategies. However, efficient delivery of therapeutic genes to relevant inner ear structures remains a major challenge for clinical translation. In this study, we evaluated the inner ear transduction profile of AAV8BP2, an engineered AAV8-derived capsid, in comparison with AAV2.7m8 and AAV8 in mice. Neonatal mice received posterior semicircular canal (PSCC) injections at postnatal day 0 (P0), and viral transduction in the cochlea and ES was assessed at P7. To examine age-dependent differences in viral transduction, additional experiments were performed in adult mice injected at P21 and analyzed at P28. We also compared three surgical delivery routes for inner ear gene transfer: PSCC injection, round window membrane (RWM) injection, and RWM injection combined with PSCC fenestration. AAV8BP2 effectively transduced the ES in both neonatal and adult mice and showed greater GFP expression in the spiral prominence than AAV8 following neonatal administration. Among the three delivery routes evaluated in adult mice, PSCC injection achieved the highest ES transduction while maintaining cochlear hair cell transduction comparable to that achieved with RWM-based approaches. Together, these findings define the relative transduction profiles of the tested AAV capsids and delivery routes and provide a basis for selecting vector-delivery route combinations for *SLC26A4*-targeted inner ear gene therapy.

## 1. Introduction

Hearing loss is one of the most common sensory disorders worldwide and represents a major public health concern [1,2]. Genetic factors contribute substantially to congenital and early-onset hearing loss, and more than 150 genes have been identified as causative factors for hereditary deafness [3,4,5,6]. Among these genes, *SLC26A4*, which encodes the anion transporter pendrin, is one of the most frequently mutated genes associated with hereditary hearing loss and is responsible for disorders such as Pendred syndrome and DFNB4, both of which are characterized by fluctuating hearing loss and vestibular dysfunction [7,8,9]. Pendrin is expressed in epithelial tissues of the inner ear and plays an essential role in regulating anion transport and maintaining endolymph homeostasis, particularly within the endolymphatic sac (ES) [10,11,12,13]. Disruption of pendrin function alters ionic balance in the inner ear and leads to progressive hearing impairment [14,15,16]. Although considerable progress has been made in understanding pendrin-associated hearing loss, no curative therapy has yet been developed [17,18,19].

Recent advances in adeno-associated virus (AAV)-mediated gene therapy have created new opportunities for treating genetic hearing loss [19,20,21]. AAV vectors are widely used for gene delivery because of their favorable safety profile, ability to transduce non-dividing cells, and capacity for long-term gene expression [22,23,24,25,26]. However, efficient gene delivery to all relevant inner ear structures remains challenging, particularly in the ES, a key site of pendrin expression. Several engineered AAV capsids have therefore been developed to improve gene transfer efficiency in target tissues. For instance, AAV2.7m8 has demonstrated efficient transduction of both hair cells and non-sensory supporting cells in the cochlea [27,28]. Anc80L65, a synthetic AAV capsid reconstructed from predicted ancestral sequences, has also shown broad and efficient transduction of cochlear hair cells and supporting cells [29]. AAV8BP2 was developed through directed selection of an AAV8 capsid library in which residues 587–595 of the capsid protein were randomized. The selected variant, containing the nine-amino-acid sequence PERTAMSLP in this region, showed enhanced transduction of retinal ON-bipolar cells compared with parental AAV8 [30]. In the inner ear, AAV8BP2 has been reported to efficiently transduce the cochlear lateral wall and ES in mice [31].

In addition to viral vector properties, the route of viral delivery plays a critical role in determining the efficiency and safety of gene transfer in the inner ear [28]. Several surgical approaches have been developed for inner ear gene delivery, including posterior semicircular canal (PSCC) injection and round window membrane (RWM) injection, each with distinct advantages and limitations [32,33]. AAV8BP2 transduces the neonatal ES with high efficiency [31], and delivery route influences cochlear hair cell transduction in adult mice [33]; however, no study has directly examined the effect of delivery route on AAV8BP2-mediated ES transduction, nor whether this tropism is preserved with advancing age. In the present study, we evaluated the inner ear transduction efficiency of AAV8BP2 in comparison with AAV2.7m8 and AAV8 in neonatal and adult mouse models. Furthermore, we investigated how different surgical delivery routes influence viral transduction efficiency in the cochlea and ES. Through this comparative analysis, we aimed to identify an optimized viral vector and delivery strategy for pendrin-targeted inner ear gene therapy.

## 2. Results

### 2.1. Location of the Engineered Peptide Sequence in the AAV8 VP3 Capsid Protein

**Figure 1:**
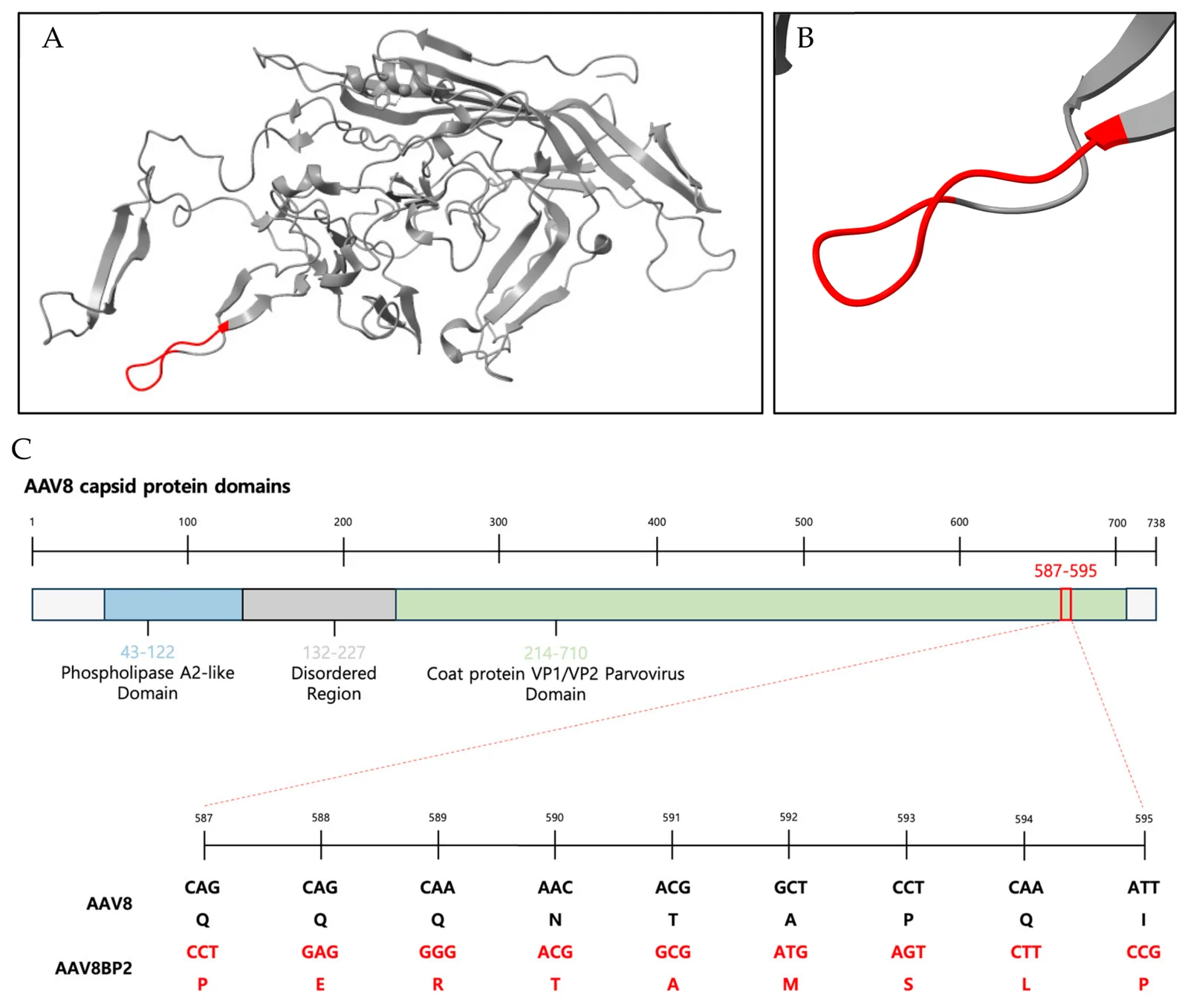
Structural location of the engineered peptide sequence in the AAV8 VP3 capsid protein. (**A**) Three-dimensional structure of the AAV8 VP3 capsid protein. The location corresponding to the engineered peptide sequence in AAV8BP2 is highlighted in red. (**B**) Close-up view of the peptide sequence site on the surface-exposed loop of the AAV8 VP3 capsid protein. (**C**) Schematic representation of the VP3 protein domains and amino acid sequence comparison between AAV8 (WT) and the engineered AAV8BP2 capsid, illustrating the previously reported nine-amino-acid peptide (PERTAMSLP) sequence at residues 587–595 of VP3.

The location of the engineered peptide sequence was mapped onto the three-dimensional structure of the AAV8 VP3 capsid protein. The randomization site is located on a surface-exposed loop of the VP3 protein (Figure 1A,B) [30]. Sequence comparison between AAV8 and AAV8BP2 showed that AAV8BP2 contains a nine-amino-acid peptide (PERTAMSLP) at residues 587–595 of the VP3 protein, while the remaining VP3 sequence is conserved (Figure 1C).

### 2.2. AAV8BP2 Exhibits ES Transduction Comparable to AAV8 in Neonatal Mice

**Figure 2:**
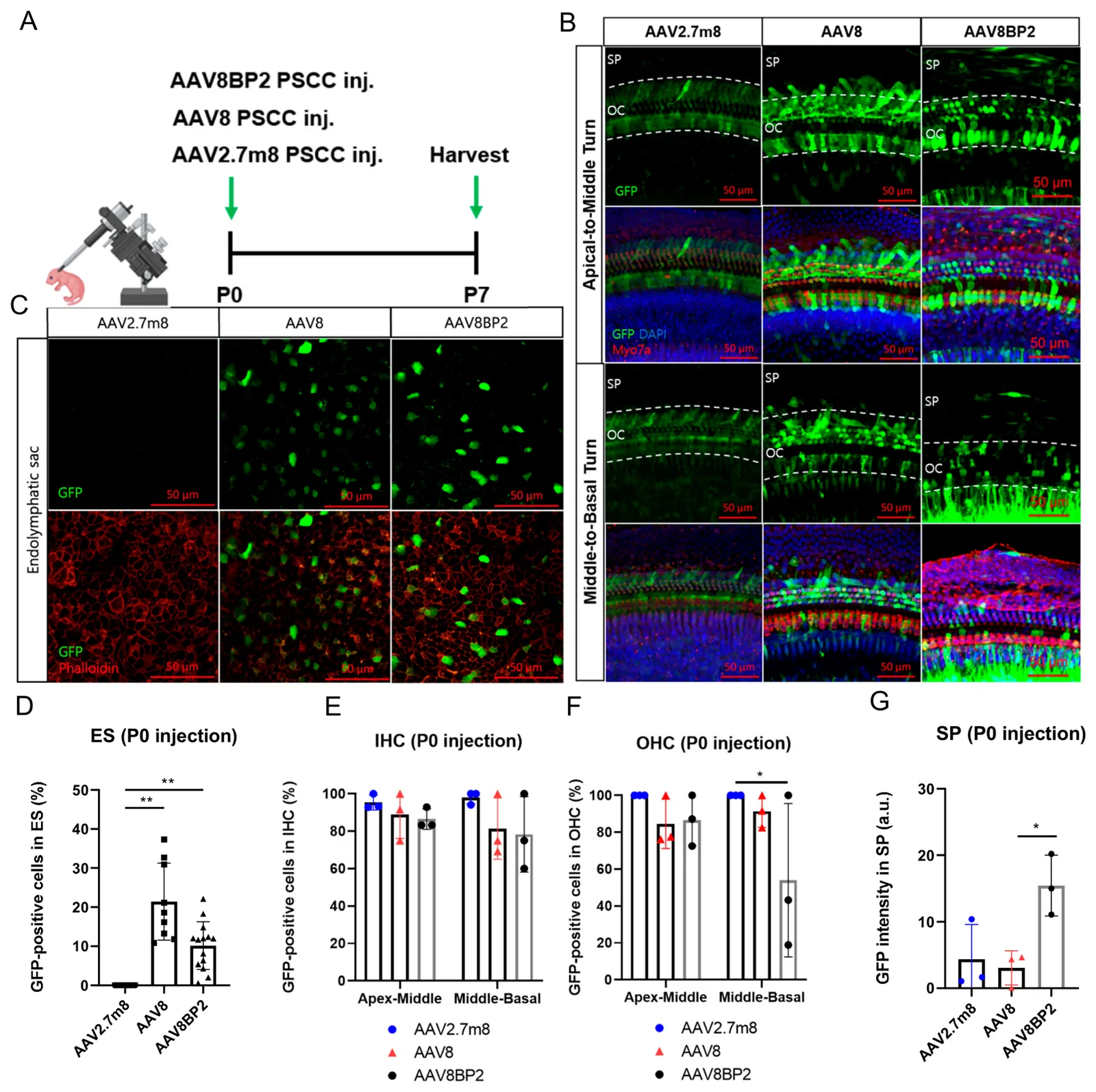
Comparison of inner ear transduction efficiencies of AAV2.7m8, AAV8, and AAV8BP2 in neonatal mice. (**A**) Experimental design. Neonatal mice received posterior semicircular canal (PSCC) injections of EGFP-expressing AAV2.7m8, AAV8, or AAV8BP2 at postnatal day 0 (P0), and inner ears were harvested at P7. (**B**) Representative fluorescence images of GFP expression in the organ of Corti (OC) and the spiral prominence (SP). GFP, green; MYO7A, red; DAPI, blue. Images were obtained from the apical and basal turns. (**C**) Representative fluorescence images of GFP expression in the endolymphatic sac (ES). GFP, green; phalloidin, red. (**D**) Quantification of GFP-positive cells in the ES. AAV8 (n = 9), AAV2.7m8 (n = 9), and AAV8BP2 (n = 14). (**E**) Quantification of inner hair cell (IHC) transduction efficiency in the apical and basal turns, expressed as the percentage of GFP-positive/MYO7A-positive IHCs relative to the total number of MYO7A-positive IHCs within each 100-µm region of interest. AAV8 (n = 3), AAV2.7m8 (n = 3), and AAV8BP2 (n = 3) (**F**) Quantification of outer hair cell (OHC) transduction efficiency in the apical and basal turns, expressed as the percentage of GFP-positive/MYO7A-positive OHCs relative to the total number of MYO7A-positive OHCs within each 100-µm region of interest. AAV8 (n = 3), AAV2.7m8 (n = 3), and AAV8BP2 (n = 3) data are presented as mean ± SD. (**G**) Quantification of GFP fluorescence intensity in the SP of the cochleae following P0 PSCC injection. Hair cell transduction efficiencies were analyzed using two-way ANOVA with Tukey’s multiple comparisons test, whereas ES transduction efficiencies were analyzed using the Kruskal–Wallis test with Dunn’s multiple comparisons test. Inj., injection; * *p* < 0.05; ** *p* < 0.01.

To compare viral transduction efficiency in the neonatal inner ear, AAV2.7m8, AAV8, and AAV8BP2 expressing EGFP were injected into the PSCC of neonatal mice at postnatal day 0 (P0). Inner ear tissues were harvested at P7 and analyzed by immunofluorescence (Figure 2A). Robust GFP expression was observed in the organ of Corti following administration of all three vectors (Figure 2B). GFP-positive cells were detected in both the apical and basal turns, indicating efficient transduction of cochlear hair cells. No obvious differences in overall cochlear transduction patterns were observed among the three vectors. In the ES, GFP-positive cells were rarely detected following AAV2.7m8 administration, whereas both AAV8- and AAV8BP2-treated mice exhibited substantial GFP expression throughout the ES epithelium (Figure 2C). Quantitative analysis confirmed significantly higher ES transduction in the AAV8 and AAV8BP2 groups compared with the AAV2.7m8 group, while no significant difference was observed between AAV8 and AAV8BP2 (Figure 2D). Quantification of GFP-positive inner hair cells (IHCs) showed comparable transduction efficiencies among the three vectors in both the apical and basal turns (Figure 2E). Similarly, outer hair cell (OHC) transduction was largely comparable, although AAV8BP2 exhibited reduced transduction in the basal turn compared with AAV2.7m8 (Figure 2F). In addition, GFP fluorescence intensity was quantified in the spiral prominence of the cochleae, where pendrin expression is important to maintain hearing function in adult mice [10]. As a result, AAV8BP2 exhibited significantly greater GFP fluorescence intensity than AAV8 (Figure 2G).

### 2.3. Comparative Inner Ear Transduction of AAV2.7m8, AAV8, and AAV8BP2 in Adult Mice

**Figure 3:**
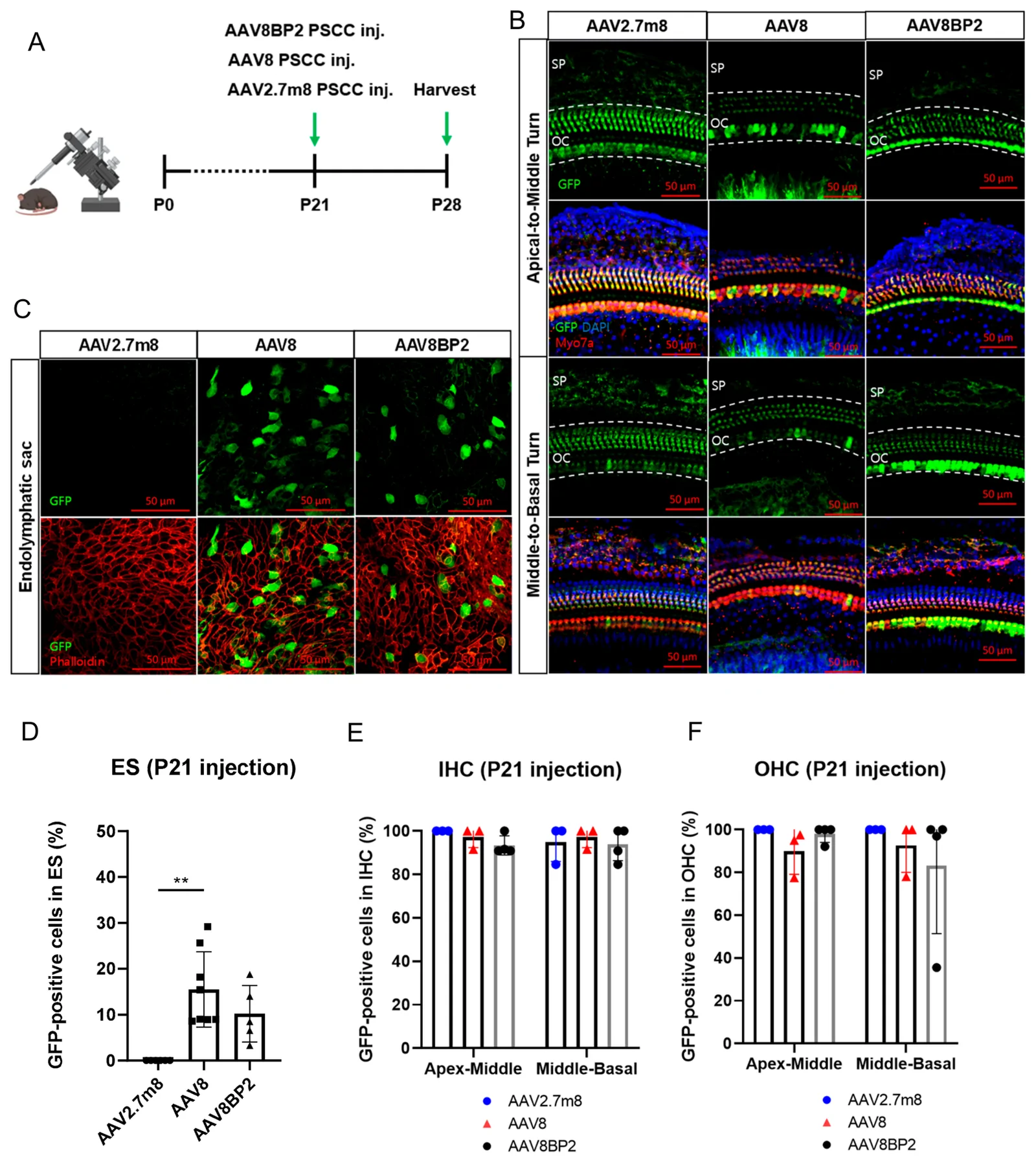
Comparison of inner ear transduction efficiencies of AAV2.7m8, AAV8, and AAV8BP2 in adult mice. (**A**) Experimental design. Adult mice received posterior semicircular canal (PSCC) injections of EGFP-expressing AAV2.7m8, AAV8, or AAV8BP2 at postnatal day 21 (P21), and inner ears were harvested at P28. (**B**) Representative fluorescence images of GFP expression in the organ of Corti (OC) and the spiral prominence (SP). GFP, green; MYO7A, red; DAPI, blue. Images were obtained from the apical and basal turns. (**C**) Representative fluorescence images of GFP expression in the endolymphatic sac (ES). GFP, green; phalloidin, red. (**D**) Quantification of GFP-positive cells in the ES. AAV8 (n = 6), AAV2.7m8 (n = 8), and AAV8BP2 (n = 5). (**E**) Quantification of inner hair cell (IHC) transduction efficiency in the apical and basal turns, expressed as the percentage of GFP-positive/MYO7A-positive IHCs relative to the total number of MYO7A-positive IHCs within each 100-µm region of interest. AAV8 (n = 3), AAV2.7m8 (n = 3), and AAV8BP2 (n = 4). (**F**) Quantification of outer hair cell (OHC) transduction efficiency in the apical and basal turns, expressed as the percentage of GFP-positive/MYO7A-positive OHCs relative to the total number of MYO7A-positive OHCs within each 100-µm region of interest. AAV8 (n = 3), AAV2.7m8 (n = 3), and AAV8BP2 (n = 4). Data are presented as mean ± SD. Hair cell transduction efficiencies were analyzed using two-way ANOVA with Tukey’s multiple comparisons test, whereas ES transduction efficiencies were analyzed using the Kruskal–Wallis test with Dunn’s multiple comparisons test. Inj., injection; ** *p* < 0.01.

To determine whether AAV8BP2 efficiently transduces the mature inner ear, AAV2.7m8, AAV8, and AAV8BP2 expressing EGFP were injected into the PSCC of adult mice at P21. Inner ear tissues were harvested seven days later and analyzed by immunofluorescence (Figure 3A). In the cochlea, GFP-positive hair cells were observed in both the apical and basal turns following administration of all three vectors (Figure 3B). Although the spiral prominence was retained in some adult specimens, it was not consistently preserved across samples during cochlear whole-mount preparation. Therefore, quantitative comparison of spiral prominence transduction among the three vector groups was not feasible. GFP expression was observed in the spiral prominence of some adult specimens, including those administered AAV2.7m8; however, inconsistent preservation of this region precluded quantitative comparisons among vectors or assessment of age-dependent differences. In the ES, GFP expression was limited in the AAV2.7m8 group, whereas GFP-positive cells were observed following AAV8 and AAV8BP2 administration (Figure 3C). Quantitative analysis revealed significantly higher ES transduction with AAV8 than with AAV2.7m8. AAV8BP2 showed an intermediate mean with greater variability, with no statistically significant differences compared with either AAV2.7m8 or AAV8 (Figure 3D). Quantification of GFP-positive IHCs and OHCs further demonstrated comparable cochlear transduction efficiencies among the three vectors in the adult mice’s cochlea (Figure 3E,F).

### 2.4. PSCC Injection Provides the Highest Transduction Efficiency in the ES

**Figure 4:**
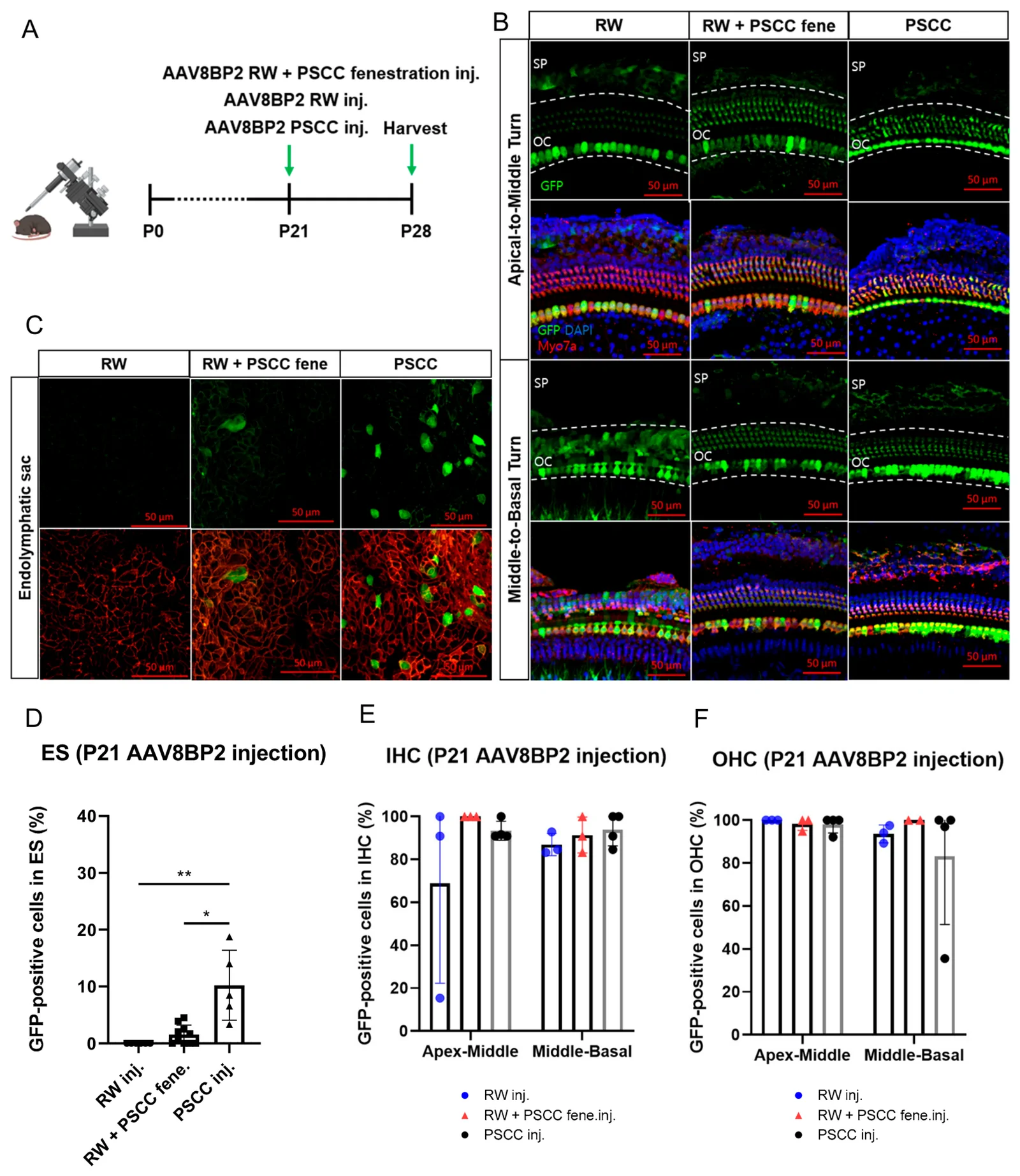
Comparison of surgical delivery routes for AAV8BP2-mediated gene transfer in the adult inner ear. (**A**) Experimental design. Adult mice received EGFP-expressing AAV8BP2 via posterior semicircular canal (PSCC) injection, round window membrane (RWM) injection, or RWM injection combined with PSCC fenestration. Inner ears were harvested at P28. (**B**) Representative fluorescence images of GFP expression in the organ of Corti (OC) and the spiral prominence (SP) following each surgical delivery route. GFP, green; MYO7A, red; DAPI, blue. (**C**) Representative fluorescence images of GFP expression in the endolymphatic sac (ES) following each surgical delivery route. GFP, green; phalloidin, red. (**D**) Quantification of GFP-positive cells in the ES. R.W inj. (n = 6), R.W inj.+ fenestration (n = 10), and PSCC inj. (n = 5) (**E**) Quantification of inner hair cell (IHC) transduction efficiency in the apical and basal turns, expressed as the percentage of GFP-positive/MYO7A-positive IHCs relative to the total number of MYO7A-positive IHCs within each 100-µm region of interest. R.W inj. (n = 3), R.W inj.+ fenestration (n = 3), and PSCC inj. (n = 4) (**F**) Quantification of outer hair cell (OHC) transduction efficiency in the apical and basal turns, expressed as the percentage of GFP-positive/MYO7A-positive OHCs relative to the total number of MYO7A-positive OHCs within each 100-µm region of interest. R.W inj. (n = 3), R.W inj.+ fenestration (n = 2), and PSCC inj. (n = 4) Data are presented as mean ± SD. Hair cell transduction efficiencies were analyzed using two-way ANOVA with Tukey’s multiple comparisons test, whereas ES transduction efficiencies were analyzed using the Kruskal–Wallis test with Dunn’s multiple comparisons test. Inj., injection; * *p* < 0.05; ** *p* < 0.01.

To compare the efficiency of different surgical delivery routes for inner ear gene transfer, AAV8BP2 expressing EGFP was administered to adult mice via PSCC injection, RWM injection, or RWM injection combined with PSCC fenestration. Inner ear tissues were harvested seven days after injection and analyzed by immunofluorescence (Figure 4A). GFP expression was observed in the organ of Corti following all three delivery methods (Figure 4B). GFP-positive hair cells were detected in both the apical and basal turns regardless of the injection route, and overall cochlear transduction patterns were comparable among the three groups. In contrast, distinct differences were observed in the ES. PSCC injection resulted in widespread GFP expression throughout the ES epithelium, whereas RWM injection produced only limited GFP-positive cells. The RWM with PSCC fenestration group exhibited an intermediate level of GFP expression between the PSCC and RWM groups (Figure 4C). Quantitative analysis demonstrated that PSCC injection achieved the highest ES transduction efficiency among the three delivery routes (Figure 4D). Although the RWM injection with PSCC fenestration group showed higher ES transduction than the RWM group, the difference was not statistically significant. Quantification of GFP-positive IHCs and OHCs further demonstrated comparable cochlear transduction efficiencies among the three surgical approaches (Figure 4E,F). Overall, among the delivery routes evaluated, PSCC injection achieved the highest ES transduction efficiency (Figure 4D).

## 3. Discussion

In the present study, we evaluated the transduction efficiency of the engineered AAV8BP2 vector in both neonatal and adult mouse inner ears and compared its performance with AAV8 and AAV2.7m8. In neonatal mice, AAV8BP2 and AAV8 achieved significantly higher ES transduction than AAV2.7m8, with no significant difference between AAV8BP2 and AAV8. In adult mice, however, only AAV8 showed significantly higher ES transduction than AAV2.7m8, whereas AAV8BP2 showed an intermediate mean with greater variability. In addition, comparison of three surgical delivery routes revealed that posterior semicircular canal (PSCC) injection produced the highest level of ES transduction while maintaining cochlear transduction comparable to the other delivery approaches. Collectively, these findings indicate that both vector selection and surgical delivery route are important determinants of efficient gene transfer to the ES.

Although remarkable advances have recently been achieved in inner ear gene therapy, most studies have primarily focused on improving transduction of cochlear sensory hair cells because these cells represent the therapeutic targets for many forms of hereditary deafness [22,28,33,34]. However, this strategy may not be sufficient for disorders caused by *SLC26A4* mutations. Pendrin, encoded by *SLC26A4*, is predominantly expressed in the epithelial cells of the ES, where it regulates endolymphatic ion transport and fluid homeostasis [7,10]. Loss of pendrin function disrupts endolymph homeostasis, leading to enlargement of the vestibular aqueduct and progressive hearing loss characteristic of Pendred syndrome and DFNB4 [7,10,35]. Accordingly, successful gene replacement therapy for *SLC26A4* requires efficient vector delivery to the ES rather than exclusively targeting sensory hair cells. Previous therapeutic studies have also demonstrated that restoration of pendrin expression can rescue hearing and vestibular phenotypes in animal models, further emphasizing the importance of efficient gene delivery to pendrin-expressing tissues [18]. Particularly, pendrin expression in the epithelia of the cochlea is crucial to maintain hearing function and prevent hearing fluctuation after birth [36].

The present study demonstrated that AAV8BP2 and AAV8 achieved comparable ES transduction efficiencies in neonatal mice. In adult mice, AAV8BP2 showed an intermediate mean with greater variability, with no significant difference observed compared with either AAV8 or AAV2.7m8. This finding is consistent with previous reports showing that AAV8 and AAV8BP2 exhibit similar transduction efficiencies in the ES epithelium following neonatal administration [31]. Notably, neonatal injection (P0–P5) has been shown to result in significantly higher transduction of marginal cells in the stria vascularis by AAV8BP2 compared with AAV8, along with a trend toward enhanced transduction of intermediate cells [31]. Consistent with these findings, our study further demonstrated that AAV8BP2 showed greater GFP expression than AAV8 in the spiral prominence following neonatal injection (Figure 2B,G). Together, these findings indicate that AAV8BP2 can transduce multiple pendrin-expressing tissues within the inner ear.

This broader tissue tropism may be particularly advantageous for *SLC26A4* gene therapy. Pendrin is expressed not only in the epithelial cells of the ES but also in the cochlear lateral wall, including the stria vascularis, where it contributes to the maintenance of endolymphatic ion homeostasis [10,18,37]. Therefore, efficient transduction of both the ES and the cochlear lateral wall may provide greater therapeutic benefit than targeting either structure alone. Our results indicate that AAV8BP2 achieves ES transduction comparable to that of AAV8, while showing higher GFP expression in the spiral prominence following neonatal administration. Together, these findings suggest that AAV8BP2 may represent a promising vector for future *SLC26A4*-targeted gene replacement strategies [10,18,31].

The distinct transduction patterns observed across capsids should be considered when developing strategies to target pendrin-expressing inner ear tissues. Both AAV8 and AAV8BP2 also transduced cochlear hair cells, indicating that their transduction was not restricted to pendrin-expressing epithelial tissues. Anc80L65 and AAV2, both of which exhibit high transduction efficiency in hair cells, did not produce detectable adverse effects when used to deliver *SLC26A4* in murine models of DFNB4 [18,19,29]. These previous findings suggest that ectopic pendrin expression in sensory hair cells may be tolerated in mouse models. Nevertheless, future vector engineering for *SLC26A4* gene therapy should prioritize efficient transduction of the ES and non-sensory epithelial cells of the cochlear lateral wall while limiting transduction of non-target cell populations.

In addition to vector selection, our results demonstrate that the route of administration influences gene delivery to the ES. Among the three surgical approaches evaluated, PSCC injection achieved the highest ES transduction while maintaining cochlear transduction comparable to that of the RWM-based approaches. Several surgical approaches, including RWM injection, cochleostomy, and PSCC injection, have been developed for inner ear gene delivery, each exhibiting distinct patterns of viral distribution and transduction efficiency depending on the target tissue [33,38,39,40,41]. Previous studies have demonstrated that PSCC injection provides broad viral distribution throughout the inner ear while preserving both auditory and vestibular function [42]. Our findings extend these observations by showing higher ES transduction with PSCC injection than with RWM injection alone or RWM injection combined with PSCC fenestration. Because the ES is anatomically continuous with the vestibular endolymphatic compartment [43], direct access through the PSCC may facilitate more efficient vector delivery to the ES than approaches relying primarily on scala tympani distribution. This experimental advantage, however, should not be interpreted as evidence of clinical superiority, as PSCC injection is not currently an established route for inner ear gene delivery and is more technically challenging and less clinically feasible than RWM injection.

Several limitations of this study should be acknowledged. First, vector performance was evaluated using GFP reporter expression rather than therapeutic *SLC26A4* gene replacement. Consequently, whether the observed transduction efficiencies are sufficient to restore pendrin expression and rescue auditory function remains to be determined. Second, GFP expression in the spiral prominence was assessed using fluorescence intensity rather than by direct quantification of GFP-positive cells because individual transduced cells could not be reliably delineated in the cochlear whole-mount preparations used in this study. Fluorescence intensity can be influenced by both the number of transduced cells and the level of reporter expression within individual cells and therefore should not be interpreted as a direct measure of cellular transduction efficiency. Accordingly, the observed differences in spiral prominence GFP intensity should be interpreted as relative differences in reporter expression rather than direct differences in cellular transduction efficiency. Third, all experiments were conducted in wild-type mice, precluding assessment of therapeutic efficacy in a disease model. Accordingly, the transduction profiles observed in this study should be interpreted as vector-distribution data rather than evidence of therapeutic efficacy in *SLC26A4*-associated hearing loss. Finally, although transduction was evaluated in both neonatal and adult mice, the long-term persistence of transgene expression was not assessed. Therefore, the present data do not establish whether the observed transduction patterns are maintained over extended periods.

Future studies should evaluate AAV8BP2-mediated *SLC26A4* gene replacement in Pendred syndrome or DFNB4 models to determine whether restoration of pendrin expression can recover endolymphatic homeostasis and preserve hearing. In addition, optimization of viral dose, injection timing, and surgical technique may further improve therapeutic efficacy and facilitate clinical translation. Together, our findings characterize the transduction profiles of AAV8BP2 in comparison with AAV8 and AAV2.7m8 and demonstrate the influence of the delivery route on ES transduction, providing a foundation for further development of gene delivery strategies targeting *SLC26A4*-associated hearing loss.

## 4. Materials and Methods

### 4.1. Animals

Wild-type C57BL/6N mice (male and female) were used in this study. Viral injections were performed at postnatal day 0 (P0) or postnatal day 21 (P21), depending on the experimental design. Animals were randomly assigned to each experimental group. The number of animals used in each experiment is indicated in the corresponding figure legends. Animals were housed under standard laboratory conditions with a 12 h light/12 h dark cycle and provided with food and water ad libitum. All animal procedures were approved by the Institutional Animal Care and Use Committee (IACUC) of Yonsei University College of Medicine (Approval No. A2023-0113, approved on 15 May 2023) and were performed in accordance with institutional guidelines.

### 4.2. Structural Visualization of the AAV8BP2 Insertion Site

The amino acid sequence of the AAV8 VP3 capsid protein was obtained from UniProt (accession no. Q8JQF8). The three-dimensional structure of the AAV8 capsid was obtained from the Protein Data Bank (PDB ID: 2QA0). The location corresponding to the engineered peptide insertion in AAV8BP2 was visualized on the AAV8 VP3 capsid structure using UCSF ChimeraX (version 1.12). Sequence comparison between AAV8 and AAV8BP2 was manually prepared based on the VP3 amino acid sequence to illustrate the engineered peptide insertion. The structural images were generated to illustrate the location of the engineered peptide insertion on the AAV8 capsid structure.

### 4.3. AAV Vectors

Recombinant AAV vectors AAV8BP2, AAV8, and AAV2.7m8, each carrying an enhanced green fluorescent protein (EGFP) reporter under the control of the CAG promoter, were used throughout this study. AAV8BP2-CAG-eGFP (1.10 × 10^13^ GC/mL) and AAV2.7m8-CAG-eGFP (9.75 × 10^12^ GC/mL) were provided by the laboratory of Dr. Wade W. Chien (National Institute on Deafness and Other Communication Disorders, National Institutes of Health, Bethesda, MD, USA) and were identical to the vectors used in previous studies. Recombinant AAV8-CAG-eGFP was commercially produced by PackGene Biotech (Houston, TX, USA) with the expression cassette ssAAV.CAG.EGFP.WPRE.SV40pA at a titer of 1.0 × 10^13^ GC/mL. All viral vectors were stored at −80 °C until use and thawed immediately before injection.

### 4.4. PSCC Injection in Neonatal Mice

Neonatal PSCC injection was performed as previously reported [44,45,46]. Neonatal mice (P0) were anesthetized by hypothermia on ice prior to surgery. After a postauricular skin incision, the left PSCC was exposed under a surgical microscope. Viral vectors were delivered into the PSCC using a pulled glass capillary connected to a Nanoliter 2000 Microinjector (World Precision Instruments, Sarasota, FL, USA). A total volume of 1 μL was injected at a rate of 0.2 μL/min. After injection, the skin incision was closed using tissue adhesive, and the pups were allowed to recover on a heating pad before being returned to their mother.

### 4.5. Inner Ear Injection in Juvenile Mice

Juvenile mice (P21) were anesthetized with an intraperitoneal injection of Zoletil (30 mg/kg) and Rompun (10 mg/kg) prior to surgery. A postauricular incision was made to expose the surgical field under a stereomicroscope. For posterior semicircular canal (PSCC) injection, viral vectors were delivered through a MicroLumen polyethylene microtube (0.39–0.5 PI × 12″ long; MicroLumen Inc., Oldsmar, FL, USA) connected to a Nanoliter 2000 Microinjector (World Precision Instruments, Sarasota, FL, USA). A total volume of 1 μL was injected at a rate of 0.2 μL/min. For round window membrane (RWM) injection, the round window niche was exposed, and viral vectors were injected through a pulled glass capillary using the same microinjection system and injection parameters. For RWM injection with PSCC fenestration, a small fenestration was created in the posterior semicircular canal using a 0.3 mL BD insulin syringe (Becton Dickinson, Franklin Lakes, NJ, USA) before viral delivery through the round window membrane. All other surgical procedures and injection parameters were identical to those used for the RWM injection group. Following injection, the incision was closed with surgical sutures, and mice were allowed to recover on a heating pad before being returned to their home cages.

### 4.6. Tissue Preparation and Immunofluorescence Staining

Inner ear immunofluorescence staining was as previously reported [47,48]. Briefly, mice were euthanized by carbon dioxide (CO_2_) inhalation, after which the cochleae and ESs were carefully dissected. Cochlear tissues were fixed in 4% paraformaldehyde (PFA) for 24 h at 4 °C, whereas ES tissues were fixed in 4% PFA for 1 h at 4 °C. Following fixation, cochlear tissues were decalcified prior to whole-mount preparation, whereas ES tissues were processed directly without decalcification. Both neonatal (P0) and juvenile (P21) cochlear samples underwent decalcification.

Whole-mount cochlear and ES tissues were permeabilized with 1% Triton X-100 and blocked with 10% donkey serum for 1 h before immunofluorescence staining. Hair cells were labeled using a rabbit anti-MYO7A antibody (1:800; Cat. No. 25-6790; Proteus Biosciences, Ramona, CA, USA), followed by an Alexa Fluor™ 568 donkey anti-rabbit IgG secondary antibody (1:1000; Cat. No. A10042; Invitrogen, Thermo Fisher Scientific, Waltham, MA, USA). F-actin was stained with phalloidin (1:200; Cat. No. P1951; Sigma-Aldrich, St. Louis, MO, USA), and nuclei were counterstained with DAPI (1:5000; Invitrogen, Thermo Fisher Scientific, Waltham, MA, USA). GFP expression was visualized by native fluorescence without antibody amplification.

### 4.7. Image Acquisition and Quantitative Analysis

Confocal images were analyzed using ImageJ (version 1.54g; National Institutes of Health, Bethesda, MD, USA). For cochlear whole-mount preparations, a 100 μm region of interest (ROI) was defined in the apical and basal turns. IHCs and OHCs positive for MYO7A were manually counted, and transduced hair cells were identified by the co-localization of GFP and MYO7A. Transduction efficiency was calculated as the percentage of GFP-positive hair cells relative to the total number of MYO7A-positive hair cells within each ROI.

For ES analysis, a 100 μm × 100 μm ROI was selected from each whole-mount preparation. The total number of DAPI-positive nuclei and GFP-positive/DAPI-positive cells within the ROI was manually counted using ImageJ. ES transduction efficiency was calculated as the percentage of GFP-positive cells relative to the total number of DAPI-positive cells. Identical image acquisition settings were maintained within each experiment, and all samples were analyzed using the same quantification criteria. Because individual GFP-positive cells within the spiral prominence could not be reliably delineated and counted in the cochlear whole-mount preparations, GFP fluorescence intensity was used as a semi-quantitative measure of relative GFP expression. The mean gray value was measured within a 10 × 100 µm rectangular region of interest (ROI) using Fiji software (ImageJ version 1.54g; National Institutes of Health, Bethesda, MD, USA). Identical image acquisition settings and ROI dimensions were applied across experimental groups to minimize variability in fluorescence measurements.

### 4.8. Statistical Analysis

All statistical analyses were performed using GraphPad Prism (version 8.0.2, GraphPad Software, Boston, MA, USA). Data are presented as mean ± standard deviation (SD). Hair cell transduction efficiencies were analyzed using two-way analysis of variance (two-way ANOVA) with Tukey’s multiple comparisons test, whereas ES transduction efficiencies were analyzed using the Kruskal–Wallis test with Dunn’s multiple comparisons test, which was considered a more appropriate and conservative approach given the non-normal distribution and floor effect observed in the data. Differences were considered statistically significant at *p* < 0.05.

## 5. Conclusions

In conclusion, AAV8BP2 achieved efficient gene delivery to the ES in both neonatal and adult mouse inner ears. Among the surgical approaches evaluated, PSCC injection provided the highest ES transduction efficiency. Collectively, these findings identify AAV8BP2 combined with PSCC injection as a promising strategy for ES-directed *SLC26A4* gene replacement therapy.

## Data Availability

The data presented in this study are available from the corresponding author upon reasonable request.

## Abbreviations

The following abbreviations are used in this manuscript:

AAV	Adeno-associated virus
ES	Endolymphatic sac
PSCC	Posterior semicircular canal
RWM	Round window membrane
IHC	Inner hair cell
OHC	Outer hair cell
ROI	Region of interest
PFA	Paraformaldehyde
GFP	Green fluorescent protein
P0	Postnatal day 0
P21	Postnatal day 21
SD	Standard deviation
ANOVA	Analysis of variance
